# Supplementary material for: Uncomfortably high: Testing reveals inflated THC potency on retail Cannabis labels
Source: PLoS One. 2023 Apr 12;18(4):e0282396. doi: 10.1371/journal.pone.0282396 (PMC10096267; doi:10.1371/journal.pone.0282396)
Supplement: S1 Table — (PDF) [file pone.0282396.s001.pdf]

Supplementary Materials for

Uncomfortably High: Testing Reveals Inflated THC Potency on Retail Cannabis Labels

Anna L. Schwabe, Vanessa Johnson, Joshua Harrelson, Mitchell E. McGlaughlin

Corresponding author. Email: Anna Schwabe: schw0701@bears.unco.edu and Mitchell McGlaughlin: Mitchell.McGalughlin@unco.edu

This PDF file includes:

HPLC data for each sample

Strain	Dispensary	Abbreviation	Sample ID	Run number	published sativa/Indica percentage	dominant strain type	MHL Total THC	**MHL average THC**	STDV	reported low	reported high
Colombian Gold '72	Good Chemistry	CoGo72_1	714	714-1	100/0	Sativa	16.52793	**12.97866**	1.9489	19.2	
				714-2			13.71213				
				714-3			12.87807				
Colombian Gold '72	Good Chemistry	CoGo72_2	887	887-1	100/0	Sativa	11.48882				
				887-2			11.61032				
				887-3			11.65468				
Sour Amnesia	Good Chemistry	SoAm_1	279	279-1	75/25	Sativa	13.33669	**15.18695**	1.7889	23.1	26.8
				279-2			12.66957				
				279-3			15.41629				
Sour Amnesia	Good Chemistry	SoAm_2	431	431-1	75/25	Sativa	16.68735				
				431-2			16.98411				
				431-3			16.02771				
Durban Poison	The Kind Room	DuPo_1SN	918	918-1	100/0	Sativa	10.47948	**11.93084**	1.3841	17.4	
				918-2			11.03936				
				918-3			13.10719				
Durban Poison	Walking Raven	DuPo_4Sn	492	492-1		Sativa	11.97292	**13.36808**	2.0276	20.88	
				492-2			13.23904				
				492-3			15.94241				
Durban Poison	Infinate Wellness	DuPo_5SN	487	487-1		Sativa	12.8844	**14.57028**	0.5749	21.5	
				487-2			13.62467				
				487-3			14.01654				
Gorilla Glue	Walking Raven	GG#4_2	189	189-1	65/35	Sativa	14.63741	**15.68007**	0.7716	25.41	30.88

				189-2			14.94816					
				189-3			15.59013					
Gorilla Glue #4	Walking Raven	GG#4_1	553	553-1	65/35	Sativa	16.61717					
				553-2			16.00701					
				553-3			16.28056					
Green Crunch (Green Crack)	Good Chemistry	GrCr_1	500	500-1	65/35	Sativa	14.62499	**14.90416**	0.5471	12.8	19.3	
				500-2			14.15312					
				500-3			14.69547					
Green Crunch (Green Crack)	Good Chemistry	GrCr_2	857	857-1	65/36	Sativa	15.34685					
				857-2			15.68819					
				857-3			14.91633					
Lemon Skunk	Good Chemistry	LeSk_2	188	188-1	60/40	Sativa	15.98159	**17.65165**	2.5673	16.9	17.4	
				188-2			15.77992					
				188-3			16.29176					
Lemon Skunk	Good Chemistry	LeSk_1	695	695-1	60/40	Sativa	22.54375					
				695-2			18.32026					
				695-3			16.99263					
OG Kush	Diego Pellicer	OGKu_1SN	794	794-1	55/45	Hybrid	15.78267	**15.00859**	0.3501	15.2	26.14	
				794-2			16.09804					
				794-3			15.39886					
OG Kush	The Kind Room	OGKu_2SN	368	368-1		Hybrid	15.52311	**15.71113**	0.2659	28.07	31.28	
				368-2			15.89916					
OG Kush	Infinate Wellness	OGKu_3SN	707	707-1		Hybrid	16.90008	**17.79265**	0.0789	24.2		
				707-2			16.91754					
				707-3			16.77292					
OG Kush	Company	OGKu_4SN	399	399-1		Hybrid	16.41026	**16.6756**	0.2754	15	25.01	
				399-2			16.65653					
				399-3			16.95999					
Mob Boss	Good Chemistry	MoBo_1SN	649	649-1	50/50	Hybrid	16.45245	**15.30407**	0.2327	19	31	
				649-2			16.55793					
				649-3			16.11255					

Mob Boss	Peak	MoBo_3SN	877	877-1		Hybrid	14.22108	**14.56471**	0.7355	22.12	24.87
				877-2			13.64287				
				877-3			15.10335				
Mob Boss	Wellness	MoBo_5SN	531	531-1		Hybrid	16.04941	**14.60793**	0.4439	25.2	28.9
				531-2			15.2305				
				531-3			15.34296				
Blue Dream	Good Chemistry	BlDr_1SN	609	609-2	50/50	Hybrid	14.4044	**14.80023**	0.7236	17.33	33
				609-3			15.42772				
Blue Dream	Lucy Sky	BlDr_3SN	409	409-1	50/50	Hybrid	17.9296	**17.47143**	0.3213	17.87	
				409-2			18.17833				
				409-3			18.5671				
Blue Dream	Peak	BlDr_4SN	445	445-1	50/50	Hybrid	12.36896	**11.16516**	0.5346	14.41	25.18
				445-2			11.94951				
				445-3			11.30749				
Blue Dream 5	Alternatives	BlDr_5_2	367	367-1	50/50	Hybrid	10.84693	**11.07359**	0.3293	16.64	
				367-2			10.31057				
				367-3			10.24767				
Blue Dream	Wellness	BlDr_6SN	738	738-1	50/50	Hybrid	15.34735	**15.71228**	1.0286	26.65	28.23
				738-2			14.06532				
				738-3			16.09956				
Bubba 98	The Kind Room	Bu98_1	372	372-1	20/80	Indica	12.64704	**16.39574**	3.2774	24.38	
				372-2			13.01693				
Bubba 98	The Kind Room	Bu98_2	412	412-1	20/80	Indica	18.20762				
				412-2			19.26065				
				412-3			18.84645				
Dankey Kong	Walking Raven	DaKo_1	178	178-1	10/90	Indica	19.67159	**19.584**	1.9205	26.58	27.73
				178-2			17.59522				
				178-3			17.31888				
Danky Kong	Walking Raven	DaKo_2	815	815-1	10/90	Indica	21.0015				
				815-2			22.29269				
				815-3			19.62409				
Afghani	The Kind Room	Afgh_1	296	296-1	0/100	Indica	9.704809	**11.27614**	1.0894	16.4	
				296-2			11.36848				

				296-3			10.40858
Afghani	The Kind Room	Afgh_2	423	423-1	0/100	Indica	12.074
				423-2			11.39194
				423-3			12.70905
