# Supplementary material for: Uncomfortably high: Testing reveals inflated THC potency on retail Cannabis labels
Source: PLoS One. 2023 Apr 12;18(4):e0282396. doi: 10.1371/journal.pone.0282396 (PMC10096267; doi:10.1371/journal.pone.0282396)
Supplement: Attachment — Submitted filename: Response_to_Reviewers.docx [file pone.0282396.s002.docx]

August 17, 2022

Dear Dr. Walker,

Thank you for the recent reviews on our manuscript PONE-D-22-138, *Uncomfortably High: Testing Reveals Inflated THC Potency on Retail Cannabis Labels*. We have addressed all of the reviewer comments, as detailed below, and submitted an updated version of the manuscript with and without track changes. Overall, we feel that the comments and our edits have strengthened the manuscript and it is ready for publication. If you have any questions or concerns, feel free to contact us.

Sincerely,

Mitchell McGlaughlin and Anna Schwabe

Please update our Funding Statement to read as follows:

“Fifteen of the samples were jointly purchased between the McGlaughlin Lab and Headspace Sensory LLC (Fort Collins, Colorado) as part of another collaborative study. All other expenses were covered by the authors.”

Academic Editor Comments

- The abstract should be more detailed and the introduction should include appropriate references of similar studies that have already been published.
- We have substantially expanded the abstract to add more background and context to our findings. We have also added more background to introduction, particularly related to previous THC potency studies (particularly LN 88-114), and expanded our literature cited throughout the manuscript.
- De-identification of the dispensaries from which product for testing was obtained unless the authors are able to establish consent from the stated dispensaries.
- All dispensary information has been de-identified.
- Expended details on testing methods both by the authors and the methods used by the dispensaries, highlighting any differences.
- We have provided additional context on how our HPLC methods relate to the testing labs our conducting. As we have now clarified, we used the method that is advertised by all current testing facilities in the state of Colorado (LN 261-265) and our analytical methods were developed based on State of Colorado testing guidelines (LN 159-160).
- Sample size justification
- We have provided more context on our sampling detailing that it was largely opportunistic (LN 126-128, 141-142). This study came about when we were collecting data for a sensory project and we discovered that there was a large discrepancy between reported and observed potency. Based on this unexpected result we validated our methods by retesting 8 samples, as detailed in the methods, and expanded our sampling as resources allowed. Although our sample size is relatively small, that in no way impacts the result that there is substantial over-reporting of potency occurring in the marketplace (LN 222-225).

It is also suggested that the term recreational cannabis be changed to non-medical use of cannabis.

- We do not like the term ‘non-medical use’, but we have replaced all references to ‘recreational’ to ‘retail” as referred to by the Colorado Department of Revenue Cannabis Enforcement Division.

Reviewer 1 Comments

Reviewer #1: This is a thoughtful paper that is well written. My only comment is that the implications for public health policy should be drawn out more fully in the discussion. What does this mean for keeping consumers in a potentially "safer" regulated and legal market? While accurate THC potency is a quality control issue, is is also a consumer trust issue and I think there would be concerns (beyond profit motives) about losing people to an illicit market?

- We have added more context related to public health in the discussion specifically addressing public trust (LN 282-288).

Finally, while this paper addresses flower product I think it requires some thought about implications for shatter and dabs - the products typically associated with the highest THC potency.

- Although the question of accuracy related to reported potency for extracts and concentrates is of interest, it is outside the scope of the current work. We did suggest that additional THC containing products should be tested in a similar study (LN 283-285) and included reference to one review paper that summarizes what is known about label accuracy for other products (LN 288).

Reviewer 2 Comments

Reviewer #2: The issue described in this manuscript, specifically the listed potency of delta-9 THC products commercially and their actual potency, is an important question and standardized testing or regulations around testing have not been formalized. While this is an important topic to study, the gaps in the literature are not well articulated given that other studies have analyzed commercially available products and have demonstrated discrepancies in listed vs. actual values

- We have expanded our introduction to cover more recent publications related to listed vs. actual potency values (LN 88-98) and reports of “lab shopping” (LN 100-114). However, this continues to be an understudied area with very limited studies that have collected empirical data.

It seems that the information should be coded to maintain confidentiality, even though these were purchased legally by investigators. Given that samples were purchased there without dispensaries consenting to research, can the authors state the ethical conduct of this study and approval by the institution?

- All dispensaries have been de-identified.

How does the testing of product completed as part of this study compare to how dispensaries may be testing product? The specifics of testing among sampled dispensaries is not listed, but some general sense of how these tests are normally being conducted would be helpful.

- We have expanded our description of how testing is normally done in Colorado and that HPLC is the most common method (LN 261-265).

The justification for 23 samples is not provided and no justification is provided for the number selected from each city and from each dispensary.

- We have provided more context on our sampling detailing that it was largely opportunistic (LN 126-128, 141-142). This study came about when we were collecting data for a sensory project and we discovered that there was a large discrepancy between reported and observed potency. Based on this unexpected result we validated our methods by retesting 8 samples, as detailed in the methods, and expanded our sampling as resources allowed. Although our sample size is relatively small, that in no way impacts the result that there is substantial over-reporting of potency occurring in the marketplace (LN 222-225). We have also reorganized Table 1 and Figure 2 so samples are grouped by dispensary and discussed that the observed discrepancies occurred for all dispensaries with 2 or more samples in the dataset (LN 220-222).
