# Supplementary material for: Uncomfortably high: Testing reveals inflated THC potency on retail Cannabis labels
Source: PLoS One. 2023 Apr 12;18(4):e0282396. doi: 10.1371/journal.pone.0282396 (PMC10096267; doi:10.1371/journal.pone.0282396)
Supplement: Attachment — Submitted filename: Response_to_Reviewers_R2.docx [file pone.0282396.s003.docx]

December 6, 2022

Dear Dr. Walker,

Thank you for the recent reviews on our manuscript PONE-D-22-13833R1, *Uncomfortably High: Testing Reveals Inflated THC Potency on Retail Cannabis Labels*. We have responded to all of the staff editor and reviewer comments, as detailed below, and submitted an updated version of the manuscript with and without track changes. Overall, we feel that the manuscript is ready for publication. If you have any questions or concerns, feel free to contact us.

Sincerely,

Mitchell McGlaughlin and Anna Schwabe

- We understand that you purchased cannabis from local dispensaries for this study. In your Methods section, please provide additional details regarding the source of this material assayed. For instance, if available, please provide the geographic coordinates and/or names of the purchase locations/dispensaries (e.g., stores, markets), as well as any further details about the purchased items (e.g., lot number, source origin, description of appearance).

To address this comment, we did two things.

1) We added dispensary license numbers and zip codes to Table 1 (Ln 225). As we previously communicated to the editorial staff (but never received a response), we do not want to list dispensaries by name because our goal was not single out ‘good’ or ‘bad’ dispensaries, rather we wanted to show that there are THC potency reporting issues across many producers. Removing dispensary names was also requested by a reviewer during the review process. We feel that the details in Table 1 now strike the appropriate balance between our goals and documenting where sampling occurred.
2) We reworked our sampling details in the methods (Ln-151-158). Since we were looking at broad patterns there was not a specific sampling strategy beyond having samples with a diversity of Sativa/Indica proportions and reported THC concentrations (ln 154-155).

We do not feel that descriptions of the material, beyond that we purchased flower, is necessary. This is particularly relevant since we approached this study via a consumer perspective. Other sample details, including lot # or source, are incredibly inconsistent from producers, making them not informative. These reporting issues are highlighted throughout our manuscript and in multiple cited studies within.

- In addition, please provide further details about the criteria used to select the dispensaries from which to assay samples.

Addressed relative to the previous requested edit. Our sampling strategy is addressed in the introduction (Ln 121-126) and methods (Ln 136-137, 141-145).

- Lastly please consider expanding on the limitations of the study, including on the generalisability of the findings for all retail Cannabis labels as inferred from the title of this work.

We previously had content that addressed our sample size limitations (Ln 244-247). In this version we have expanded upon that discussion (Ln 248-251) providing additional citations that support that our findings are consistent with what is occurring across the industry. We also added additional text to the introduction (Ln 108-112) to include a second lawsuit related to inflated THC potency and then adjusted our conclusions section (Ln 340-343) to make clear that our results are specific to samples in Colorado and to recommend further research including more samples from other states.

Reviewer Comments

Reviewer #2: Revisions are satisfactory and I recommend acceptance. Data availability is unclear though in the statement provided by the authors and should be clarified per journal requirements.

All of our data is available in supplementary Table S1 as stated on Ln 575 and uploaded into the manuscript system.

Reviewer #3: The manuscript is well written, the data are of public and scientific interest, the results interpretation is balanced and on point.
Consider adding some comments that THC concentrations could also have a different meaning depending on the potential CBD presence, concentration, and ratio.

This request is outside the scope of our manuscript. The reviewer is absolutely correct that there are different effects and descriptions based on combinations of different cannabinoids. This is an active area of study within the field, which is best approached with different methods and study design. All samples included in this study were high THC drug-type samples available at recreational *Cannabis* dispensaries. We make clear that we are looking solely at THC % throughout the introduction based on the importance of THC % to regulation/defining drug-type *Cannabis* (Ln 43-44) and the importance it plays in marketing and pricing Cannabis (Ln 74-112).
